# Fibroblast Growth Factor 2 Induces E-Cadherin Down-Regulation via PI3K/Akt/mTOR and MAPK/ERK Signaling in Ovarian Cancer Cells

**DOI:** 10.1371/journal.pone.0059083

**Published:** 2013-03-15

**Authors:** Man-Tat Lau, Wai-Kin So, Peter C. K. Leung

**Affiliations:** Department of Obstetrics and Gynecology, Child and Family Research Institute, University of British Columbia, Vancouver, British Columbia, Canada; II Università di Napoli, Italy

## Abstract

Fibroblast growth factor 2 (FGF2) is produced by ovarian cancer cells and it has been suggested to play an important role in tumor progression. In this study, we report that FGF2 treatment down-regulated E-cadherin by up-regulating its transcriptional repressors, Slug and ZEB1, in human ovarian cancer cells. The pharmacological inhibition of phosphatidylinositol-3-kinase (PI3K), mammalian target of rapamycin (mTOR), and MEK suggests that both PI3K/Akt/mTOR and MAPK/ERK signaling are required for FGF2-induced E-cadherin down-regulation. Moreover, FGF2 up-regulated Slug and ZEB1 expression via the PI3K/Akt/mTOR and MAPK/ERK signaling pathways, respectively. Finally, FGF2-induced cell invasion was abolished by the inhibition of the PI3K/Akt/mTOR and MAPK/ERK pathways, and the forced expression of E-cadherin diminished the intrinsic invasiveness of ovarian cancer cells as well as the FGF2-induced cell invasion. This study demonstrates a novel mechanism in which FGF2 down-regulates E-cadherin expression through the activation of PI3K/Akt/mTOR and MAPK/ERK signaling, and the up-regulation of Slug and ZEB1 in human ovarian cancer cells.

## Introduction

Epithelial ovarian cancer (EOC), which comprises 90% of all ovarian malignancies, is the most common and lethal form of gynecological cancer in developed countries [1], the death rate for this disease has not changed much in the last 50 years.

Fibroblast growth factor-2 (FGF2) mediates various cellular events, including proliferation, motility, and differentiation [2], [3], and malignant ovarian tumors are common in patients with elevated FGF2 [4]–[6]. However, the role of FGF2 in ovarian cancer progression is still controversial. Ovarian tumors with high cytoplasmic FGF2 are associated with reduced tumor aggressiveness and increased survival rates compared with patients with low levels of FGF2 [7], [8]. In contrast, previous *in vitro* studies and the gene expression profiling of advanced ovarian cancer suggest that FGF2 acts as an autocrine growth factor for ovarian cancer cell proliferation [9]–[11] and invasion [12]. Moreover, FGF2 regulates the expression of additional genes implicated in angiogenesis or metastasis, including metalloproteinases [13], vascular endothelial growth factor [14], and E-cadherin [13], [15], [16].

E-cadherin functions as a cell-cell adhesion protein and tumor suppressor that is silenced in many malignancies, and the loss of E-cadherin expression or function is a common event in tumor progression [17], [18]. E-cadherin is known to suppress tumor cell invasion, and the re-expression of E-cadherin in E-cadherin-deficient carcinomas reverts cells to a less invasive, less aggressive phenotype [19]–[21], while the loss of E-cadherin is associated with ovarian cancer metastasis, peritoneal dissemination, and poor prognosis [22]–[26]. The loss of E-cadherin function can be achieved by the mutation of the E-cadherin gene [27], the hypermethylation of the E-cadherin promoter [28], [29], and the transcriptional repression of E-cadherin [30]–[33]. Several transcription factors have been identified to suppress E-cadherin including Snail, Slug, Twist and ZEB1 via their interaction with the E-box binding site in the E-cadherin promoter [30], [31], [34]–[36].

Previous studies have demonstrated that FGF2 suppresses E-cadherin in various cell types [13], [15], [16]; however, the underlying mechanisms are still largely unknown. In the present study, we demonstrate that FGF2 reduces E-cadherin mRNA and protein levels in a time- and dose-dependent manner. Furthermore, increased Slug and ZEB1 expression via the activation of the PI3K/Akt/mTOR and the MAPK/ERK signaling pathways, respectively, potentially mediates the effects of FGF2 on E-cadherin. Finally, our results indicate that the down-regulation of E-cadherin-mediated FGF2 enhances the invasiveness in ovarian cancer cells.

## Materials and Methods

### Materials

FGF2 was purchased from Sigma-Aldrich (Ontario, Canada). Rapamycin, U0126 and wortmannin were purchased from Calbiochem (San Diego, CA). E-cadherin antibodies were purchased from BD Biosciences (San Jose, CA). Akt, phospho-Akt (Ser473), p44/42 MAPK (ERK), phospho-p44/42 MAPK (Thr202/Tyr204), p70S6K and phospho-p70S6K (Thr389) antibodies were purchased from Cell Signaling Technology, Inc. (Beverly, MA). The β-actin antibody was purchased from Santa Cruz Biotechnology (Santa Cruz, CA). Horseradish peroxidase-conjugated goat anti-rabbit IgG and goat anti-mouse IgG antibodies were purchased from Bio-Rad Laboratories (Hercules, CA).

### Plasmid Constructs

The pcDNA-GFP (GFP) was generously provided by Dr. Alonzo H. Ross [37]. The pcDNA-Ecadherin-GFP (Ecad-GFP) was a kind gift from Dr. Jennifer L. Stow [38].

### Cell culture and transfections

Human ovarian cancer cell lines (OVCAR-4 and SKOV-3) were purchased from the American Type Culture Collection (ATCC, Manassas, VA), and their use was approved by the University of British Columbia Clinical Screening Committee for Research and Other Studies Involving Human Subjects. Cells were cultured in Medium 199:MCDB 105 (1∶1; Sigma-Aldrich) containing 10% fetal bovine serum (FBS; Hyclone Laboratories Ltd., Logan, UT), 100 U/ml penicillin G and 100 µg/ml streptomycin (Life Technologies, Inc., Rockville, MD) in a humidified atmosphere of 5% CO_2_ to 95% air at 37°C. The cells were passaged with 0.06% trypsin (1∶250)/0.01% EDTA in Mg^2+^/Ca^2+^ - free HBSS at confluence.

All transfections were carried out using Lipofectamine 2000 Reagent (Invitrogen, Burlington, ON, Canada) according to the manufacturer's protocol.

### Reverse transcription quantitative real-time PCR (RT-qPCR)

Total RNA was prepared using TRIzol reagent (Invitrogen) according to the manufacturer's instructions. Single-stranded cDNA was synthesized from 2 µg total RNA according to the manufacturer's procedure (Amersham Biosciences, Quebec, Canada). The primers used for SYBR Green RT-qPCR were as follows: for human E-cadherin, sense, 5′-ACA GCC CCG CCT TAT GAT T-3′ and antisense, 5′-TCG GAA CCG CTT CCT TCA-3′; for Slug, sense, 5′-TTC GGA CCCACA CAT TAC CT-3′ and antisense, 5′-GCA GTG AGG GCA AGA AAA AG-3′ ; for ZEB1, sense, 5′-GCA CCT GAA GAG GAC CAG AG-3′ and antisense, 5′-TGC ATC TGG TGT TCC ATT TT-3′; and for GAPDH, sense, 5′-ATG GAA ATC CCA TCA CCA TCT T-3′ and antisense, 5′-CGC CCC ACT TGA TTT TGG -3′. RT-qPCR was performed using the Applied Biosystems 7300 Real-Time PCR System equipped with a 96-well optical reaction plate. Relative quantification of mRNA levels was performed using the comparative Cq method (ΔΔCq method) with GAPDH as the reference gene.

### Western blot analysis

Cells were harvested in lysis buffer [50 mM Tris (pH 7.5), 150 mM NaCl, 1% NP40, 0.5% sodium deoxycholate, 1 mM EDTA, 0.1% SDS] containing protease inhibitor cocktail (Sigma-Aldrich), and protein concentrations were determined using the DC Protein Assay (Bio-Rad Laboratories, Inc., Hercules, CA). Protein (40 µg) was electrophoresed on 7.5% SDS-polyacrylamide gels, transferred to nitrocellulose membranes (Amersham Bioscience), and incubated with specific primary antibodies at 4°C overnight. After washing, the membranes were incubated with horseradish peroxidase-conjugated secondary antibodies for 1 h and were visualized with enhanced chemiluminescent substrate (Thermo Fisher Scientific Inc, Waltham, MA).

### Invasion assay

Twenty-four-well transwell inserts with an 8-µm pore coated with 1 mg/ml Matrigel (50 µl/well; BD sciences, Mississauga, ON, Canada) were used to assess cell invasion. Trypsinized cells (1×10^5^) in 0.1% FBS medium, with or without FGF2, were seeded in triplicate in the upper chamber. 1% FBS medium was placed in the lower wells. The chambers were incubated for 24 h at 37°C in a 5% CO_2_ atmosphere. Cells that did not penetrate the filter were wiped off, and invaded cells on the lower surface of the filter were fixed with ice-cold methanol and stained with 0.5% crystal violet. Results are presented as the mean number of invaded cells of five fields (at 100× magnification) ± SEM from three independent experiments.

### Data analysis

All values are expressed as mean ± SEM from three to six independent experiments. Data were analyzed by a one-way ANOVA followed by Tukey's *post hoc* test using GraphPad Prism 5 (GraphPad Software, San Diego, CA). *P* <0.05 was considered statistically significant.

## Results

### Effect of FGF2 on E-cadherin expression in ovarian cancer cells

**Figure 1:**
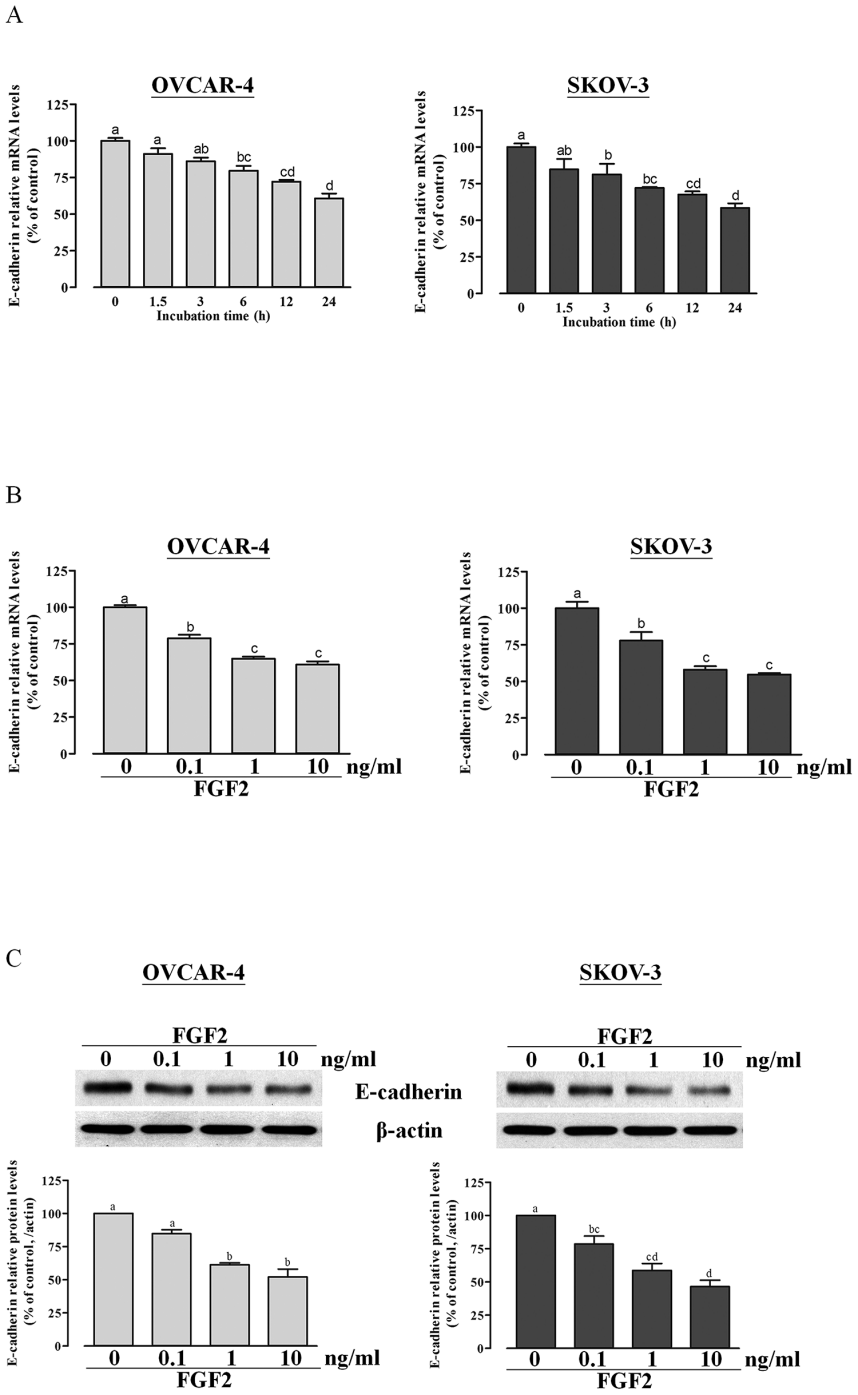
FGF2 suppresses E-cadherin mRNA and protein levels in OVCAR-4 and SKOV-3 cells. (A) OVCAR-4 and SKOV-3 cells were treated with 10 ng/ml FGF2 for 0 to 24 h as indicated, and then E-cadherin mRNA levels were analyzed by RT-qPCR. (B and C) OVCAR-4 and SKOV-3 cells were treated with different doses of FGF2 for 24 h after which E-cadherin mRNA levels (B) and protein levels (C) were analyzed by RT-qPCR and Western blotting, respectively. Results represent the mean ± SEM (n = 3; values without a common letter are significantly different, *P*<0.05). Data were analyzed by one-way ANOVA followed by Tukey's multiple comparison test.

As a first step toward analyzing the role of FGF2 in ovarian cancer progression, we investigated the effect of FGF2 on E-cadherin expression in OVCAR-4 and SKOV-3 cells. Our results showed that treatment with FGF2 down-regulated E-cadherin mRNA levels in both a time- (Fig 1A) and dose-dependent manner (Fig 1B). Similarly, Western blot analysis showed that treatment with FGF2 down-regulated E-cadherin protein levels in a dose-dependent manner in ovarian cancer cells (Fig 1C).

### FGF2-induces E-cadherin down-regulation via the PI3K/Akt and MAPK/ERK pathways

**Figure 2:**
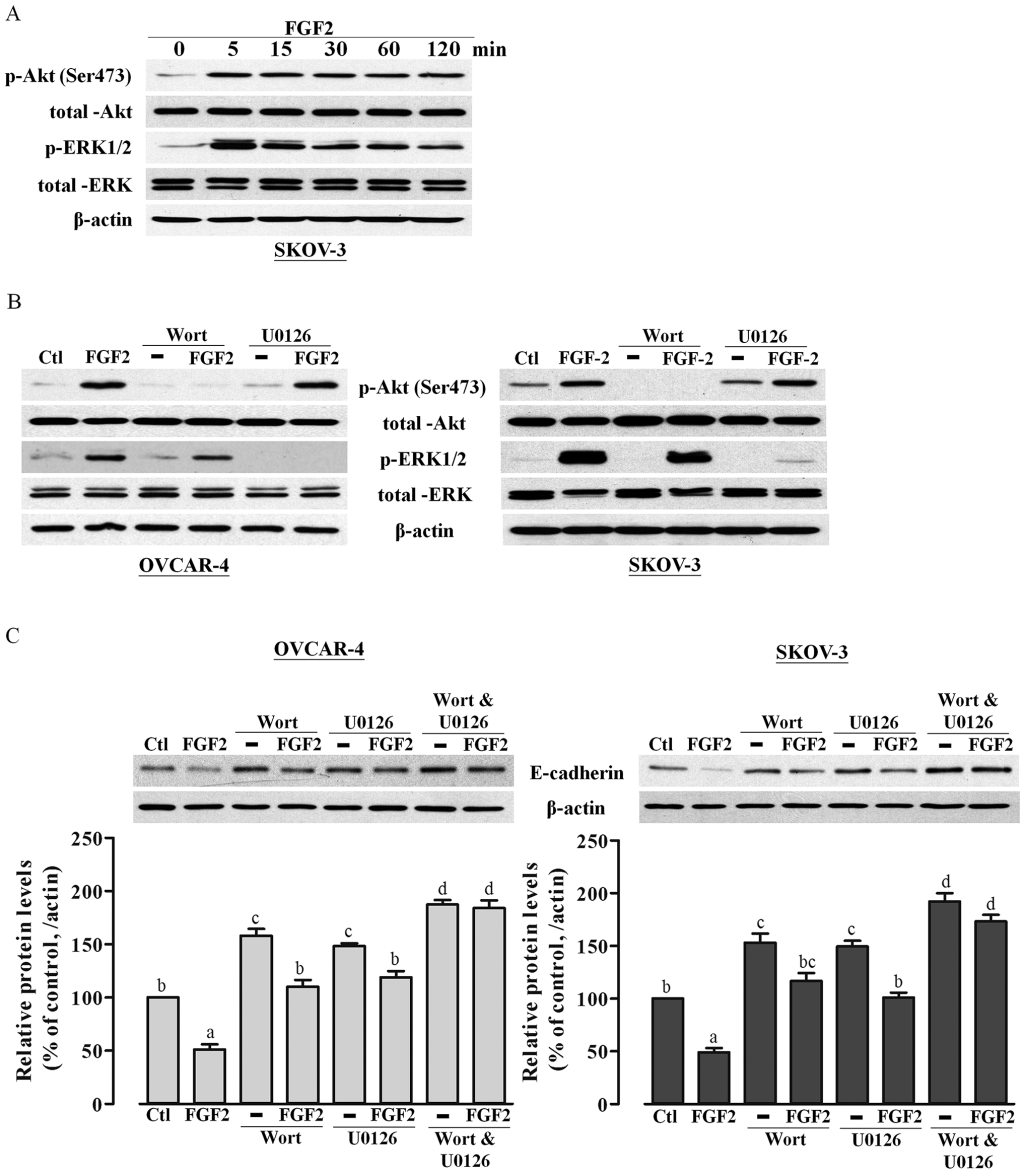
FGF2 suppresses E-cadherin expression via the PI3K/Akt and MAPK/ERK signaling pathways. (A) SKOV-3 cells were treated with 10 ng/ml FGF2 for 0 to 120 min as indicated. Phosphorylated and total Akt, phosphorylated and total ERK, and β-actin levels were analyzed by Western blot analysis. (B) OVCAR-4 and SKOV-3 cells were pretreated with wortmannin (1 µM) or U0126 (10 µM) for 30 min before the addition of FGF2 (10 ng/ml) for 30 min. Phosphorylated and total Akt, phosphorylated and total ERK protein levels were analyzed by Western blotting. The β-actin antibody was used as a control for equal loading. (C) OVCAR-4 and SKOV-3 cells were pretreated with wortmannin (1 µM) or U0126 (10 µM) alone or in the presence of 10 ng/ml FGF2 for 24 h, after which E-cadherin protein levels were analyzed by Western blotting. Results represent the mean ± SEM [(A) & (B) n = 3; (C) n = 6; values without a common letter are significantly different, *P*<0.05]. Data were analyzed by one-way ANOVA followed by Tukey's multiple comparison test.

It is well documented that the PI3K/Akt and MAPK/ERK pathways are frequently amplified and serve as survival pathways in ovarian carcinomas [39]. In addition, FGF2 is known to activate the PI3K/Akt and MAPK/ERK pathways [40], [41] and, has been reported to regulate E-cadherin down-regulation [42], [43]. Therefore, we analyzed whether these two pathways were involved in the suppression of E-cadherin expression by FGF2. We first examined the phosphorylation status of Akt and ERK upon treatment with 10 ng/ml FGF2 at 5, 15, 30, 60, and 120 minutes post-treatment and found that FGF2 treatment induced the phosphorylation of Akt (Ser473) and ERK (Thr202/Tyr204) in a time-dependent manner in SKOV-3 cells (Fig 2A). To determine whether these two pathways were involved in the suppression of E-cadherin expression by FGF2, we used two pharmacological inhibitors, wortmannin and U0126, to specifically block the PI3K/Akt and MAPK/ERK pathways, respectively, in SKOV-3 cells and OVCAR-4 (Fig 2B), respectively. As shown in Fig. 2C, the PI3K and ERK inhibitors significantly increased basal E-cadherin expression and markedly diminished, but did not completely abolish, FGF2-induced suppression of E-cadherin expression, which demonstrated the involvement of the PI3K/Akt and MAPK/ERK pathways in the FGF2-mediated down-regulation of E-cadherin expression in ovarian cancer cells.

### FGF2 differentially up-regulates Slug and ZEB1 expression via the PI3K/Akt and MAPK/ERK pathways, respectively

**Figure 3:**
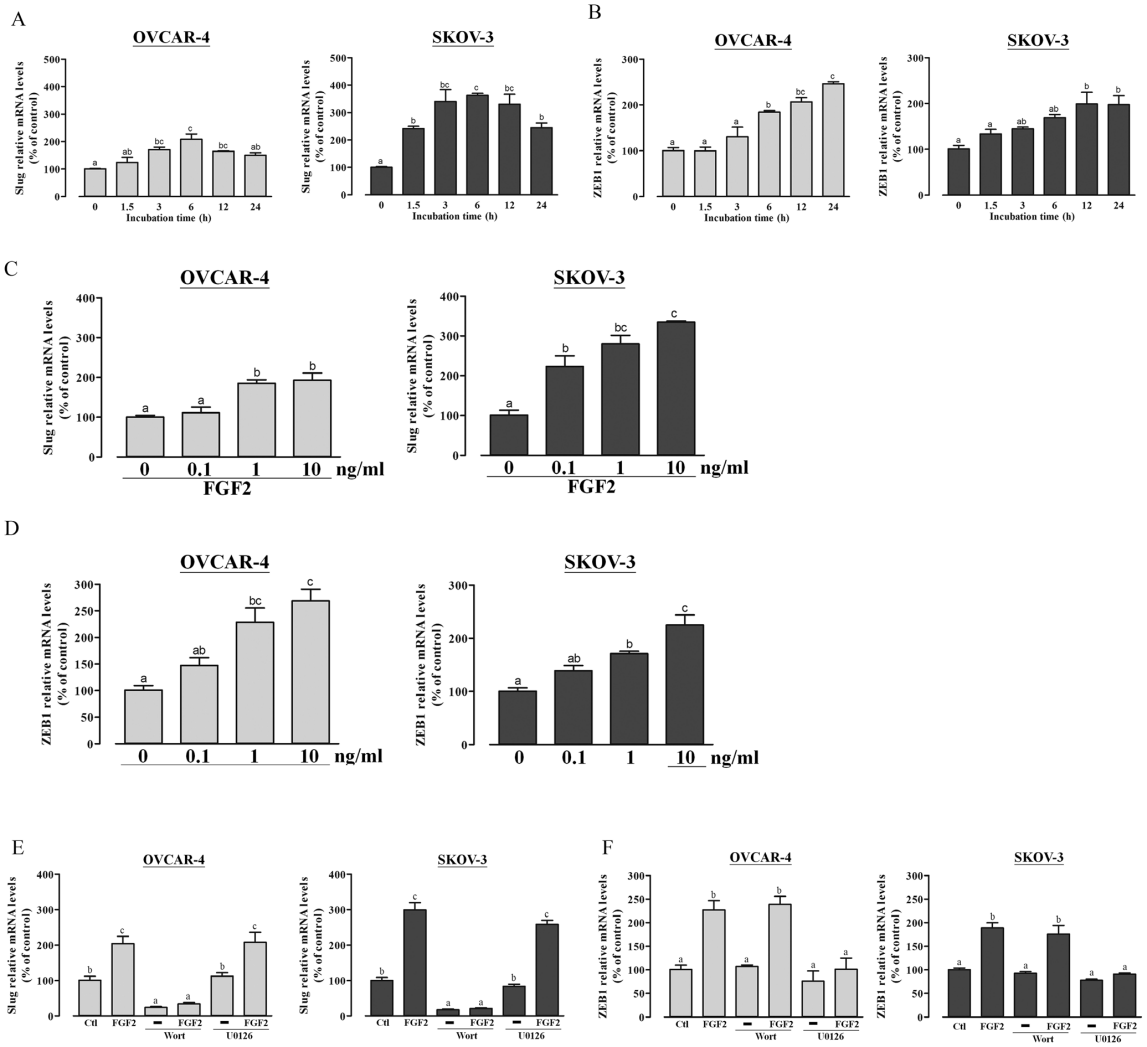
FGF2 increases Slug and ZEB1 mRNA levels in OVCAR-4 and SKOV-3 cells. (A and B) OVCAR-4 and SKOV-3 cells were treated with 10 ng/ml FGF2 for various times, and the mRNA levels of Slug (A) and ZEB1 (B) were analyzed by RT-qPCR. (C and D) OVCAR-4 and SKOV-3 cells were treated with different doses of FGF2 for 6 h (Slug; C) or 24 h (ZEB1; D), and mRNA levels were analyzed by RT-qPCR. (E and F) OVCAR-4 and SKOV-3 cells were pretreated with wortmannin (1 µM) or U0126 (10 µM) for 30 min prior to the addition of 10 ng/ml FGF2 for 6 h (E) and 24 h (F). Slug and ZEB1 mRNA levels were analyzed by RT-qPCR. Results represent the mean ± SEM (n = 3; values without a common letter are significantly different, *P*<0.05). Data were analyzed by one-way ANOVA followed by Tukey's multiple comparison test.

To investigate whether FGF2 down-regulates E-cadherin expression by modulating the transcriptional regulation of E-cadherin, we used RT-qPCR to examine the mRNA levels of the E-cadherin transcriptional repressors Snail, Slug, Twist and ZEB1. Treatment with FGF2 significantly increased Slug and ZEB1 mRNA levels in a time- (Fig 3A and 3B) and dose-dependent manner (Fig 3C and 3D) but had no significant influence on Snail and Twist mRNA levels (data not shown). To determine whether the PI3K/Akt and MAPK/ERK signaling pathways are involved in FGF2-induced increases in Slug and ZEB1 mRNA, cells were treated with PI3K inhibitor (wortmannin) or MEK inhibitor (U0126) in the presence or absence of FGF2. Interestingly, wortmannin significantly suppressed the basal Slug mRNA level and totally abolished the effects of FGF2 on Slug mRNA levels (Fig 3E), whereas FGF2-enhanced ZEB1 mRNA levels were not affected (Fig 3F). On the other hand, U0126 treatment totally abolished the effects of FGF2 on ZEB1 mRNA levels (Fig 3F), the drug had no effect on Slug mRNA levels (Fig 3E).

### FGF2-induced E-cadherin down-regulation via the PI3K/Akt/mTOR pathway

**Figure 4:**
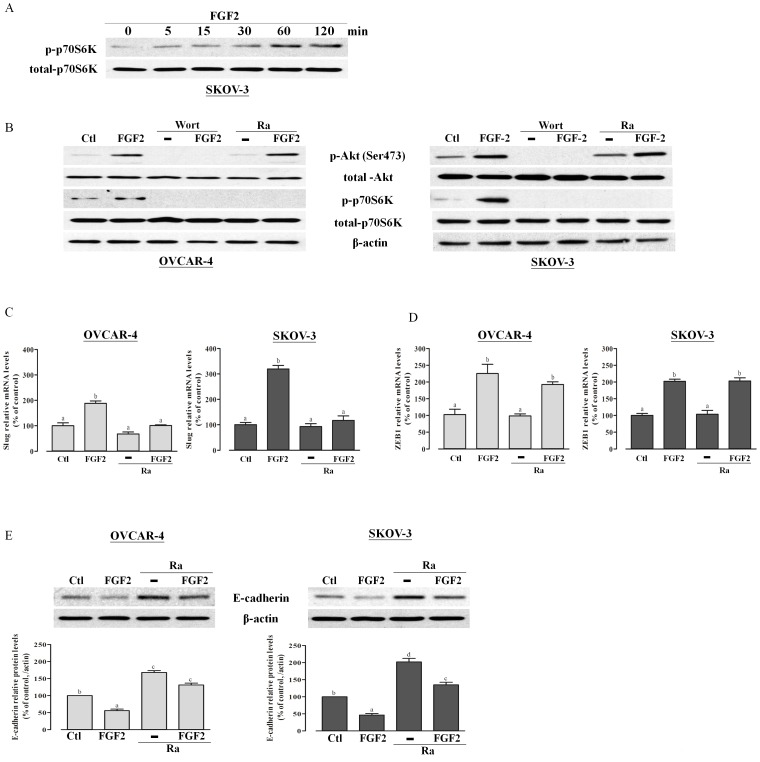
FGF2 suppresses E-cadherin expression via the PI3K/Akt/mTOR signaling pathway. (A) SKOV-3 cells were treated with 10 ng/ml FGF2 for 0 to 120 min as indicated. Phosphorylated and total p70S6K levels were analyzed by Western blot analysis. (B) OVCAR-4 and SKOV-3 cells were pretreated with wortmannin (1 µM) or rapamycin (20 nM) for 30 min prior to the addition of 10 ng/ml FGF2 for 30 min. Phospho-Akt and Akt, along with phospho-p70S6K and p70S6K protein levels were analyzed by Western blotting. The β-actin antibody was used as a control for equal loading. (C and D) OVCAR-4 and SKOV-3 cells were pretreated with rapamycin (20 nM) for 30 min prior to addition of 10 ng/ml FGF2 for 6 h (C) and 24 h (D). Slug and ZEB1 mRNA levels were analyzed by RT-qPCR. (E) OVCAR-4 and SKOV-3 cells were pretreated with rapamycin (20 nM) for 30 min prior to addition of 10 ng/ml FGF2 for 24 h, after which E-cadherin protein levels were analyzed by Western blotting. Results represent the mean ± SEM [(A)–(D) n = 3; (E) n = 6; values without a common letter are significantly different, *P*<0.05). Data were analyzed by one-way ANOVA followed by Tukey's multiple comparison test.

Next, we investigated the role of the mTOR pathway in FGF2-induced E-cadherin down-regulation, because mTOR is a pathway downstream of PI3K/Akt signaling that has been shown to be involved in E-cadherin down-regulation [44]. As shown, treatment with FGF2 induced the activation of mTOR signaling in a time-dependent manner in SKOV-3 cells, as indicated by phosphorylation of the mTOR downstream molecule, p70S6K (Fig 4A). To determine whether the PI3K/Akt/mTOR signaling pathway was involved in the regulation of E-cadherin levels by FGF2, we used the mTOR-specific inhibitor rapamycin to block the mTOR pathway in SKOV-3 cells and OVCAR-4 (Fig 4B). Similar to wortmannin, rapamycin totally abolished the FGF2-induced elevation of Slug mRNA (Fig 4C), whereas rapamycin showed no effect on ZEB1 mRNA levels (Fig 4D). Moreover, rapamycin treatment significantly increased the protein levels of E-cadherin and markedly diminished the suppressive effect of FGF2 on E-cadherin protein levels (Fig 4E), indicating that the PI3K/Akt/mTOR pathway is involved in FGF2-induced E-cadherin suppression in ovarian cancer cells.

### Activation of the PI3K/Akt/mTOR and MAPK/ERK signaling pathways are critical for FGF2-induced cell invasion

**Figure 5:**
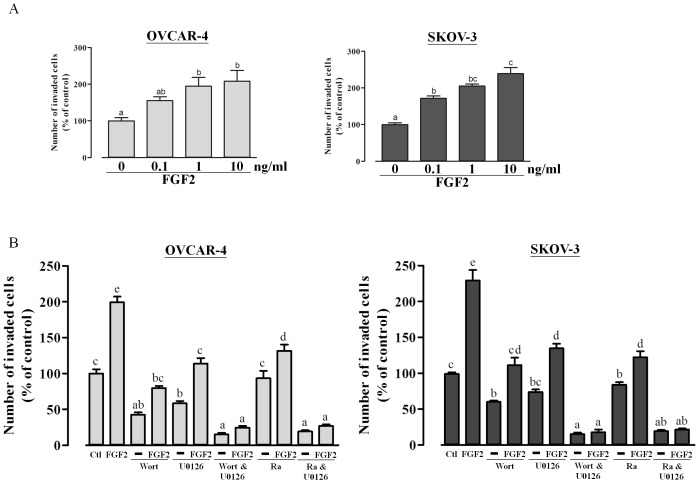
FGF2 induces ovarian cancer cell invasion via the PI3K/Akt/mTOR and MAPK/ERK signaling pathways. (A) Ovarian cancer cells were seeded in Matrigel-coated transwell inserts and treated with different doses of FGF2 for 24 h. (B) Cells were pre-treated with wortmannin (1 µM), rapamycin (20 nM) or U0126 (10 µM) for 30 min and seeded in Matrigel-coated transwell inserts and cultured with 10 ng/ml FGF2 for 24 h. Results represent the mean ± SEM [(A) n = 3; (B) n = 6; values without a common letter are significantly different, *P*<0.05]. Data were analyzed by one-way ANOVA followed by Tukey's multiple comparison test.

Several lines of evidence indicate that FGF2 plays an important role in the invasive properties of human cancer cells [45], [46]. Thus, we examined the effect of FGF2 on cell invasion in ovarian cancer cells using Matrigel-coated Transwell invasion assays. OVCAR-4 and SKOV-3 cells were treated with increasing concentrations of FGF2, resulted in a dose-dependent stimulation of invasion (Fig 5A). The involvement of the PI3K/Akt/mTOR and MAPK/ERK signaling pathways in FGF2-stimulated cell invasion were also evaluated. Our results showed that FGF2-induced cell invasion was markedly diminished, but not totally, by treatment with wortmannin, rapamycin or U0126 alone (Fig 5B), while combined inhibition of the PI3K/Akt/mTOR and MAPK/ERK signaling pathways totally abolished FGF2-induced cell invasion (Fig 5B). Taken together, these results indicate that the MAPK/ERK and PI3K/Akt/mTOR pathways are involved in FGF2-induced ovarian cancer cell invasion.

### Down-regulation of E-cadherin mediates FGF2-stimulated ovarian cancer cell invasion

**Figure 6:**
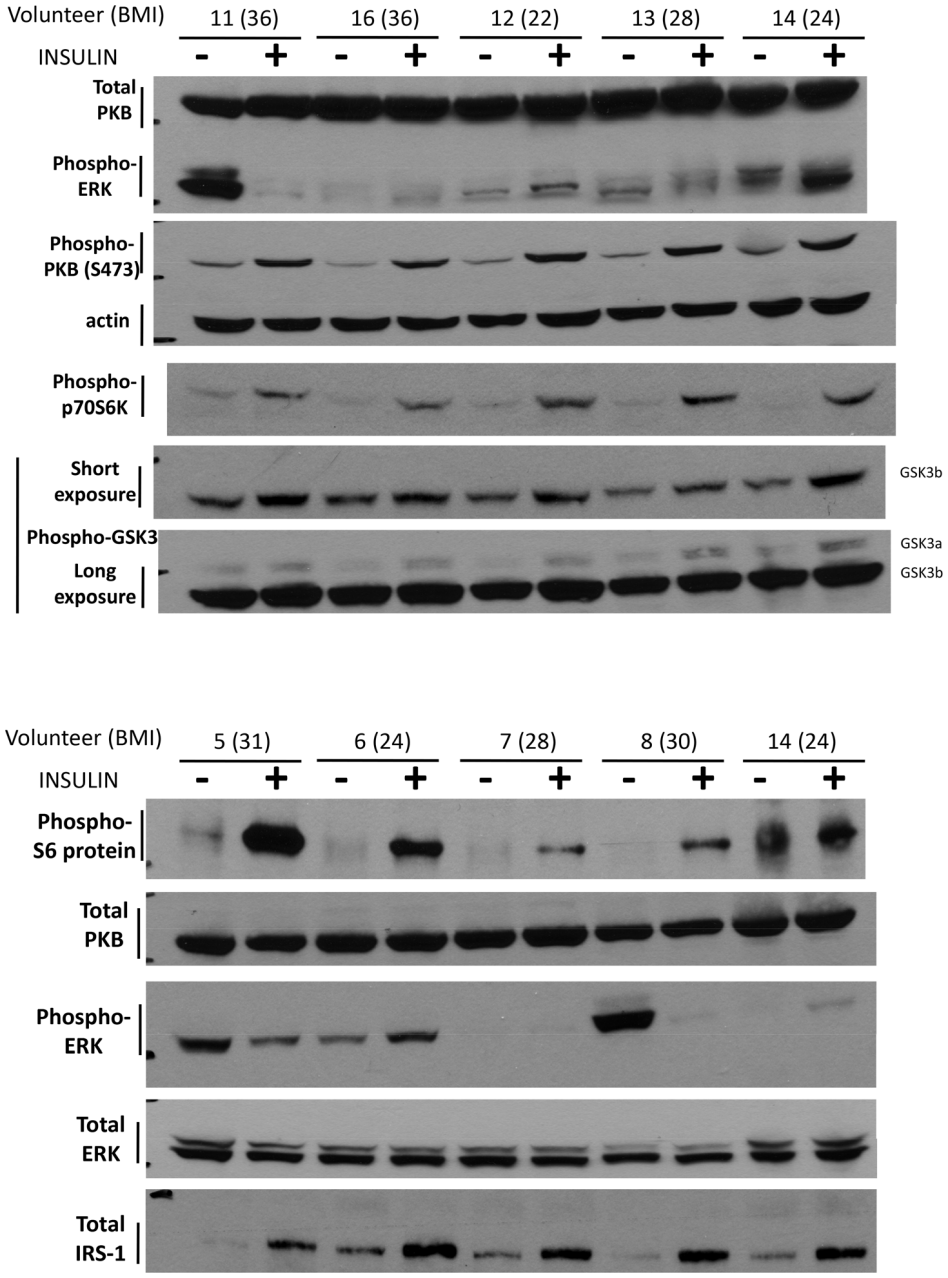
The loss of E-cadherin mediates FGF2-induced invasion. (A) SKOV-3 cells were transient transfected with the pcDNA-GFP (GFP) or human E-cadherin expression plasmids (Ecad-GFP) for 48 h. After transfection, cells were treated with 10 ng/ml FGF2 for 24 h and subjected to immunoblotting for E-cadherin and β-actin. (B) After 48 h of transfection, the trypsinized cells were seeded in Matrigel-coated transwell inserts, and cultured with 10ng/ml FGF2 for 24 h. Results represent the mean ± SEM (n = 6; values without a common letter are significantly different, *P*<0.05). Data were analyzed by one-way ANOVA followed by Tukey's multiple comparison test.

Next, we asked whether down-regulation of E-cadherin mediated the FGF2-induced cell invasion. SKOV-3 cells were transiently transfected with wild-type human E-cadherin expression plasmid for 48 h and then were treated with FGF2 for further 24 h (Fig 6A). FGF2 treatment reduced E-cadherin protein levels in cells transfected with empty vector, whereas no effect was detected in cells overexpressing E-cadherin (Fig 6A). Overexpression of E-cadherin decreased basal invasiveness and abolished the ability of FGF2 to induce ovarian cancer cell invasion (Fig 6B), implicating that E-cadherin plays an essential role in FGF-stimulated ovarian cancer cell invasion.

## Discussion

FGF2, which is a member of the FGF family, normally presents in plasma at a concentration less than 10 pg/ml, and elevated levels (up to 6 ng/ml) can be found in ascetic fluid from ovarian cancer patients [4]. Clinical studies have shown a high levels of FGF2 and its mRNA in advanced primary ovarian cancers when compared to normal ovaries [6]. Also, several studies have shown increased plasma levels of FGF2 in ovarian cancer patients [4], [47], [48]. The elevated levels of FGF2 and its receptors present in ovarian malignant tumors suggest that FGF2 plays an important role in ovarian tumor progression [9]. Several *in vitro* studies and the gene expression profiling studies in advanced ovarian cancer reveal that FGF2 functions as an autocrine growth factor for ovarian cancer cell proliferation [9]–[11], and invasion [12]. Moreover, FGF2 regulates expression of various genes implicated in angiogenesis or metastasis [13]–[15], [49], suggesting the FGF2 signaling may be a potential therapeutic target. However, the role of FGF2 in ovarian tumor progression remains to be elucidated. The present study shows that FGF2 induced the down-regulation of E-cadherin expression, which is involved in FGF2-induced ovarian cancer cell invasion. In addition, our studies suggest that FGF2 exerts its effects in human ovarian cancer cells via the activation of the PI3K/Akt/mTOR and MAPK/ERK signaling pathways and the subsequent increased expression of Slug and ZEB1.

FGF2 plays a fundamental role in various biological activities, including cell proliferation, migration, and differentiation [2], [3]. FGF2 has also been described as an angiogenic factor [50] and more recently has been shown to contribute to the development of peritioneal metastasis [51]. Dysregulated FGF signaling is common in many cancers including ovarian cancer [4]–[6], [52], suggesting that this signaling can promote tumor development and progression. FGF2 has been reported to modulate E-cadherin expression in a variety of cell types. However, its role on E-cadherin expression seems to be cell type specific. In pancreatic adenocarcinoma, FGF-1 and FGF2 have been shown to up-regulate E-cadherin expression [53]. In contrast, the down-regulation of E-cadherin expression with FGF2 treatment has been observed in tubular epithelial cells and NBT-II carcinoma cells, resulting in increased cell migration and invasion [13], [15]. Also, FGF2 has been shown to down-regulate E-cadherin expression in human umbilical vein endothelial cells via the JNK signaling pathway [16]. The current data support a crucial role for FGF2 in the down-regulation of E-cadherin in ovarian carcinoma cells via the PI3K/Akt/mTOR and MAPK/ERK signaling pathways. The binding of FGF2 to its receptors leads to the activation of downstream signaling pathways such as PI3K/Akt and MAPK/ERK [40], [41]. Emerging evidence suggests that these pathways are involved in the regulation of E-cadherin [42], [43]. Moreover, the aberrant inhibition of the mTOR pathway, which is downstream of PI3K/Akt signaling blocked FGF2-induced E-cadherin down-regulation and cell invasion. These results are consistent with our previous study demonstrating a requirement for mTOR signaling in insulin-like growrh factor 1-induced E-cadherin down-regulation in ovarian cancer cells [54]. Taken together, our results indicate that the FGF2-dependent PI3K/Akt/mTOR and MAPK/ERK activation is involved in FGF2-induced E-cadherin down-regulation and cell invasion in ovarian cancer cells.

The loss of E-cadherin gene expression is mainly due to an overexpression of transcriptional repressors including Snail, Slug and ZEB1 [34]–[36]. Indeed, elevated Slug and ZEB1 mRNA levels have been found in ovarian carcinoma [55], [56]. Moreover, a previous study demonstrated that the overexpression of Slug in SKOV-3 cells results in the down-regulation of E-cadherin, enhanced motility and invasiveness [57]. However, much less is known about the regulation of these transcriptional repressors. Slug expression can be regulated by PI3K/Akt signaling, which can also be activated by FGF treatment [40], [41], [43], [45]. Consistent with these results, the inhibition of PI3K/Akt signaling by wortmannin reduced the basal and abolished FGF2-induced Slug levels expression, suggesting that this pathway is critical for Slug expression. The activation of PI3K/Akt signaling has been demonstrated to stimulate Slug expression via GSK-3β/β-catenin signaling and to subsequently down-regulate E-cadherin in uterine carcinosarcomas and normal hepatocytes [43], [58]. It is well known that PI3K/Akt signaling induces nuclear β-catenin accumulation through the inhibition of GSK3β [59], [60]. In addition, the inhibition of mTOR signaling by rapamycin blocks increased β-catenin translocation into the nucleus in human pancreatic β-cells [61]. FGF2 may activate PI3K/Akt signaling and its downstream GSK3β and mTOR pathways to induce β-catenin-dependent transcription including that of Slug. Further studies are required to elucidate the precise mechanism of the elevation of Slug levels by the mTOR pathway. Notably, E-cadherin protein levels were clearly increased by rapamycin, while basal Slug mRNA levels were not affected. Thus, our results suggest that the mTOR signaling pathway also modulates the E-cadherin levels in Slug-independent manner. Interestingly, treatment with the MEK inhibitor U0126 only blocked FGF2-induced ZEB1 expression, but did not inhibit FGF2-induced Slug expression. Our data suggest that MAPK/ERK is an upstream factor of ZEB1 activation in ovarian cancer cells *in vitro*. FGF2 has been shown to activate the MAPK/ERK pathway in various cancer cells [62], [63], and we show in OVCAR-4 and SKOV-3 cells that ZEB1 expression is MAPK/ERK-dependent. These results are consistent with a previous study demonstrating a requirement for MAPK/ERK signaling in IGF-1-induced ZEB1 expression in prostate cancer cells [42]. Furthermore, a recent study demonstrated that ERK2, but not ERK1, reduced E-cadherin levels via Fra1-mediated ZEB1/2 in a nontransformed human mammary epithelial cell line, MCF-10A cells [64]. Taken together, our findings indicate that FGF2 differentially regulates Slug and ZEB1 expression via the PI3K/Akt/mTOR and MAPK/ERK signaling pathways in human ovarian cancer cells.

The biological significance of E-cadherin reduction in ovarian cancer invasiveness was demonstrated by the fact that overexpression of E-cadherin blocked FGF2-induced cell invasion *in vitro*. Evidence indicates that the loss of E-cadherin is associated with ovarian cancer metastasis, peritoneal dissemination and poor patient survival [22]–[26], suggesting that E-cadherin functions as a suppressor of tumor invasiveness. Indeed, silencing E-cadherin by siRNA enhances ovarian cancer cell invasion via an up-regulation of the α5-integrin [24]. We have also found that E-cadherin knockdown by RNA interference increases the PI3K/Akt signaling pathway [65], which, in turns, mediates E-cadherin-depletion-induced invasion in ovarian cancer cells (Lau *et al*., unpublished). Moreover, the overexpression of a dominant-negative E-cadherin mutant in ovarian carcinoma cells results in increased mesenchymal cell migration [66]. Our results demonstrate that FGF2 enhances cell invasiveness by down-regulating E-cadherin and that E-cadherin overexpression inhibits basal invasiveness and abolishes FGF2-induced invasion. The PI3K/Akt and MAPK/ERK signaling pathways are involved in FGF2-induced cell invasion [45], [46]. Furthermore, additional mechanisms such as the elevation of protease activity/secretion, the modulation of actin cytoskeleton, and increased motility have also been described [12], [46], [67]. Whereas loss of E-cadherin has been shown to stimulate MMP-mediated invasion in prostate cancer and bronchial tumor cells [68], [69]. Taken together, these results demonstrate that E-cadherin acts as a crucial suppressor of ovarian cancer invasiveness, and along with other described mechanisms, the loss of E-cadherin plays an important role in FGF2-induced cell invasion.

**Figure 7:**
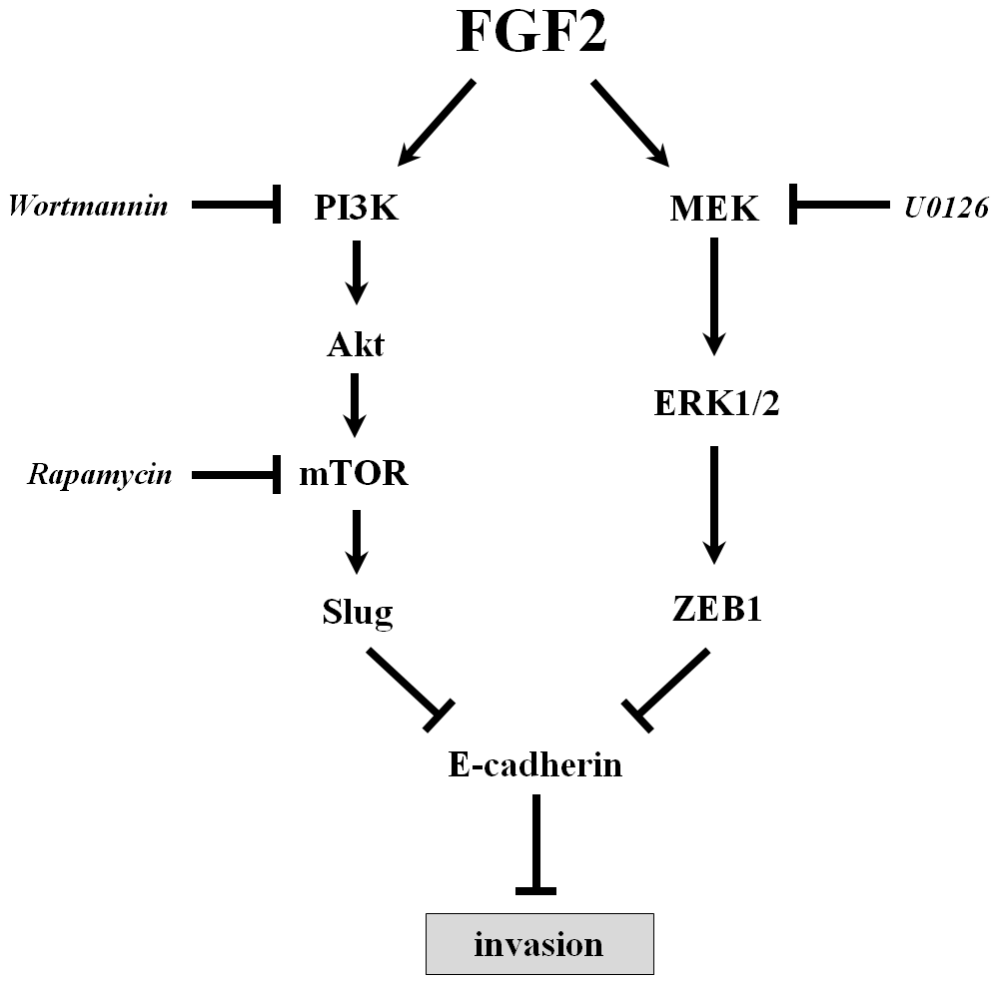
Proposed model illustrating how FGF2 suppressed E-cadherin may contribute to ovarian cancer cell invasion.

In summary, our results show that FGF2 down-regulates E-cadherin expression, most likely through the transcriptional suppression of Slug and ZEB1, which are concomitantly expressed by the activation of the PI3K/Akt/mTOR and MAPK/ERK pathways. In addition, the present study suggests that the down-regulation of E-cadherin mediates FGF2-induced ovarian cancer cell invasion (Fig 7). Inhibition of either PI3K/Akt/mTOR or MAPK/ERK signaling results in partially blocked the FGF2-induced E-cadherin down-regulation and cell invasion. Thus, these findings indicate that the design of combined treatments targeting FGF2-related signaling cascades may have relevant implications in the prevention and treatment of this malignancy.
