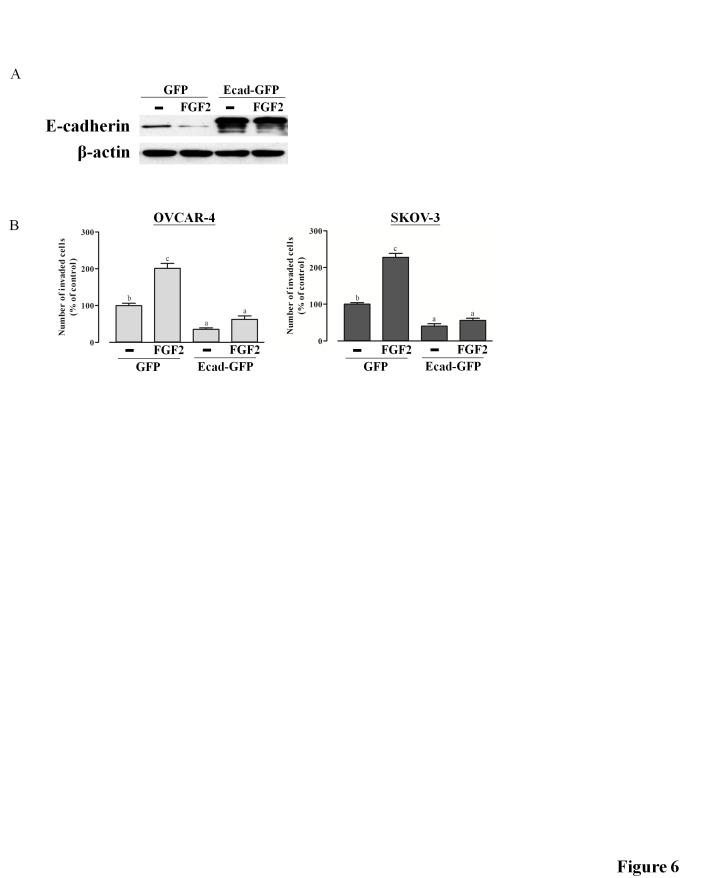# Correction: Fibroblast Growth Factor 2 Induces E-Cadherin Down-Regulation via PI3K/Akt/mTOR and MAPK/ERK Signaling in Ovarian Cancer Cells

**DOI:** 10.1371/annotation/ee15c511-4f60-4415-a0f4-c99dcf1bb2e2

**Published:** 2013-12-17

**Authors:** Man-Tat Lau, Wai-Kin So, Peter C. K. Leung

Figure 6 is the incorrect figure. Please see the correct figure 6 here: 

**Figure:**